# Diagnostic Challenges of Early T-cell Precursor Acute Lymphoblastic Leukemia: A Case Report and Literature Review

**DOI:** 10.7759/cureus.71615

**Published:** 2024-10-16

**Authors:** Mohammed Bensalah, Abdelilah Berhili, Mounia Slaoui, Assya Khermach, Rachid Seddik

**Affiliations:** 1 Hematology, Faculty of Medicine and Pharmacy of Oujda, Mohammed First University, Oujda, MAR; 2 Hematology, Mohammed VI University Hospital, Oujda, MAR

**Keywords:** cytogenetic, etp-all, flow cytometry, immunophenotyping, t-all

## Abstract

Early T-cell precursor acute lymphoblastic leukemia (ETP-ALL) is an uncommon subtype of T-cell acute lymphoblastic leukemia (T-ALL), constituting approximately 10-13% of T-ALL cases in childhood and 5-10% in adults. A 55-year-old female patient presented with no significant medical history and was hospitalized in the internal medicine department due to anemia and deterioration of her overall health. The clinical examination did not reveal any tumor syndrome. A complete blood count was performed showing normocytic normochromic anemia with leukocytosis. The myelogram revealed bone marrow invasion by blasts of 80%. Immunophenotyping revealed the presence of a blast population of T lymphoid nature estimated at 74% of the marrow elements with a particular immunophenotype. Thus, the diagnosis of ETP-ALL was retained. A karyotype was carried out showing the presence of a hypotetraploid clone with clonal anomalies in number and structure. The patient was put on hyper-CVAD protocol (cyclophosphamide, doxorubicine, vincristine, dexamethasone). A myelogram at the end of induction revealed the presence of 45% blasts.

The categorization of ETP-ALL as a distinct entity in the WHO classification arises from its distinguishable phenotypic and genetic characteristics. However, the diagnosis and management of ETP-ALL always constitutes a challenge for the biologist and clinician. Therefore, it is essential to conduct more extensive studies for a better comprehension of the clinical and biological features of ETP-ALL, specifically focusing on its genetic nuances.

## Introduction

T-cell acute lymphoblastic leukemia (T-ALL) is a highly aggressive hematologic malignancy, representing approximately 15% of acute lymphoblastic leukemia cases in children and 25% in adults. Early T-cell precursor acute lymphoblastic leukemia (ETP-ALL) is a rare subtype of T-ALL. It constitutes a distinct entity in the 2017 revision edition of the “WHO Classification of Tumors of Hematopoietic and Lymphoid Tissues”. It represents approximately 10% to 13% of cases of T-ALL in children and 5% to 10% of cases of ALL in adults [1,2]. This hemopathy is characterized by a specific immunophenotypic profile first identified by Coustan-Smith et al., as it expresses myeloid markers and hematopoietic stem cell markers in addition to T lymphoid markers [3]. Moreover, several studies have concluded that this entity presents a heterogeneous mutational status without a specific genetic abnormality. Indeed, the genetic abnormalities found in ETP-ALL are typically seen in acute myeloid leukemia (AML); on the other hand, those observed in typical T-ALL are rarely detectable or even absent [3]. These phenotypic and genotypic characteristics complicate the diagnosis and management of this entity. This hematologic disorder is associated with a poor response to standard chemotherapy and a substantially increased risk of relapse compared to other subtypes of T-ALL.

## Case presentation

**Figure 1:**
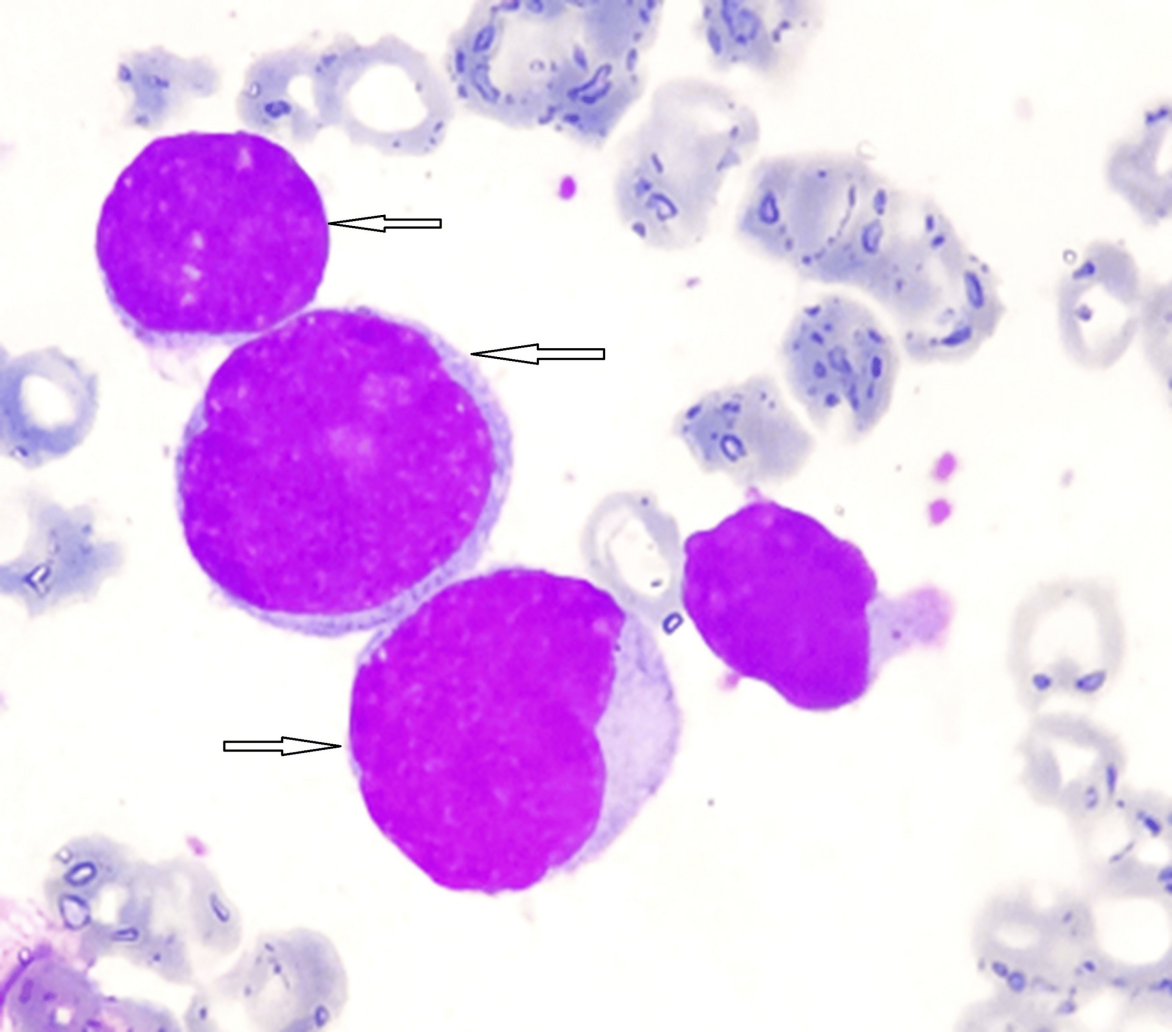
Bone marrow aspirate smear showing blasts (May-Grünwald Giemsa stain).

**Figure 2:**
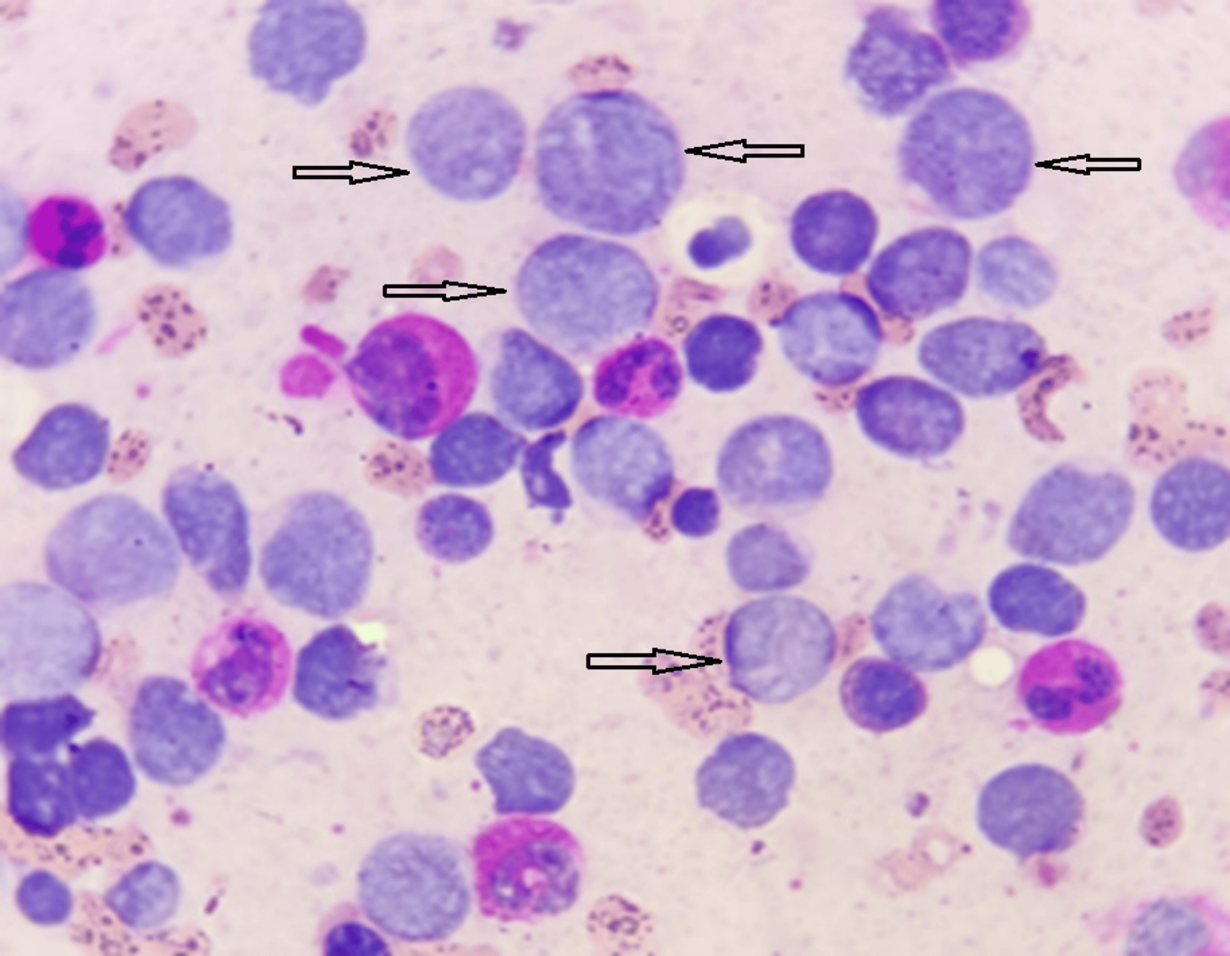
Bone marrow aspirate smear showing blasts (Myeloperoxidase stain).

**Figure 3:**
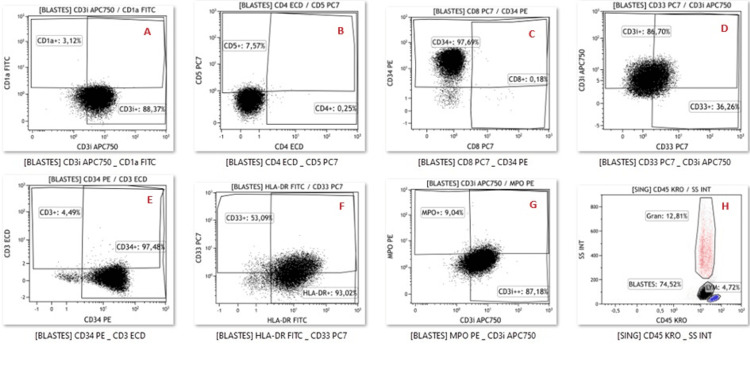
Dot plot showing the immunophenotypic profile of our ETP-ALL case. Lym: lymphocytes; Gran: granulocytes; A: expression of cytoplasmic CD3 and lack of expression of CD1a; B: lack of expression of CD5 and CD4; C: expression of CD34 and lack of expression of CD8; D: expression of CD33 and cytoplasmic CD3, E: expression of CD34 and lack expression of cytoplasmic CD3; F: expression of HLA-DR and CD33; G: lack expression of MPO and expression of CD33; H: SSC and CD45 characteristics of leukemic cells analyzed ETP-ALL: early T-cell precursor acute lymphoblastic leukemia

A 55-year-old patient with no significant medical history was admitted to the internal medicine department for treatment of anemic syndrome and deterioration of her general condition. The clinical examination was unremarkable (no signs of tumor syndrome). A complete blood count was performed showing normocytic, normochromic anemia (hemoglobin (Hb): 8.2g/dL) with leukocytosis at 60.6 g/L. The blood smear revealed a blast rate of 80%, requiring a myelogram to be performed, which revealed bone marrow invasion by blasts of 80%. Myeloperoxidase (MPO) cytochemistry was negative (Figures 1-2). Immunophenotyping revealed the presence of a blast population of T lymphoid nature estimated at 74% of the marrow elements, which expressed the immaturity markers CD34 and HLA-DR. Blasts expressed cytoplasmic CD3 but were negative for surface CD3. Intracytoplasmic MPO was also negative (the positivity threshold used is 28%). Other T lymphoid markers (CD5, CD8, CD4 and CD1a) were negative. Furthermore, we note an aberrant expression of CD33. The rest of the myelomonocytic markers (CD13, CD117, CD64, CD16, CD36) as well as the B lymphoid markers (CD19, CD22, CD10) were negative (Figure 3). Thus, the diagnosis of ETP-ALL was retained. A karyotype was carried out showing the presence of a hypotetraploid clone with clonal anomalies in number and structure: trisomies for chromosomes 4, 19 and 20, monosomies for chromosomes 1, 8, 9, 10, 11, 12, 15, 16 and 17 as well as marker chromosomes of unknown origin.

Biochemical tests revealed normal uric acid levels at 56.40 mg/L and total bilirubin at 2.80 mg/L, with direct bilirubin at 1.00 mg/L. The calcium level was 105 mEq/L, and the total protein was 66 g/L. Potassium measured 4.6 mEq/L, sodium was 139 mEq/L, and gamma-glutamyl transferase (GGT) was 30 UI/L. C-reactive protein (CRP) was significantly elevated at 115.20 mg/L, with an alkaline reserve of 25 mmol/L, and blood urea was noted at 0.23 g/L.

The patient was put on a hyper-CVAD protocol (cyclophosphamide, doxorubicine, vincristine, dexamethasone). A myelogram at the end of induction revealed the presence of 45% blasts (induction failure). After this, the patient was considered a candidate for chimeric antigen receptor (CAR) T-cell therapy but unfortunately passed away before the treatment could be administered.

## Discussion

Diagnosis of acute leukemia (AL) is based on morphological, phenotypic and genetic characteristics. Indeed, immunophenotypic analysis is required to study the expression of surface and cytoplasmic antigens in order to classify AL as myeloid or B or T lymphoid. This analysis follows the demonstration of more than 20% bone marrow and/or blood blasts [1], although this criterion is no longer retained in WHO 2022, and most AML with recurrent genetic abnormalities can be diagnosed with a blast rate of less than 20% [2]. However, in some cases of so-called lineage-ambiguous AML, the immunophenotypic profile is not characteristic of a lineage. This category includes a wide range of immature AL that either do not express lineage-specific antigens or on the contrary, express a combination of lymphoid and myeloid antigens. These include AL of ambiguous lineage with defining genetic abnormalities and AL of ambiguous lineage, immunophenotypically defined [2]. AL with minimal differentiation may also be one of the leukemias whose diagnosis is based on immunophenotyping.

T-ALL is an aggressive hematological malignancy that represents approximately 15% of cases of all acute lymphoblastic leukemia in children and 25% in adults [1]. ETP-ALL is a rare form of T-ALL, representing roughly 10-13% of cases in children and 5-10% in adults [1]. It was first described in 2009 by Coustan-Smith et al. (2009) based on its particular immunophenotypic profile (expression of lymphoid, myeloid and stem cell markers) and a genetic profile similar to that of normal ETP cells, a subpopulation of thymocytes that retain a potential for multilineage differentiation [3]. It is a separate provisional entity in the 2017 Revision Edition of the “WHO Classification of Tumors of Hematopoietic and Lymphoid Tissues”. It was also included in the new WHO 2022 classification. This hemopathy is characterized by a poor response to conventional chemotherapy and a very high risk of relapse in comparison with the other subtypes of T-ALL [3-5]. It also presents an immunophenotypic profile and cytogenetic abnormalities similar to those found in T lymphoid/myeloid mixed phenotype acute leukemia (T/M-MPAL), which makes its diagnosis and treatment a challenge [6].

The immunophenotypic profile of this entity is characterized by the absence of expression of CD1a and CD8 (<5% of positive lymphoblasts), low expression of CD5 (with <75% of positive lymphoblasts) and expression of at least 25% of lymphoblasts of one or more myeloid markers or stem cell markers in particular: CD13, CD33, CD11b, CD65, CD34, CD117 or HLA-DR, while MPO is negative. Blasts also express cytoplasmic CD3 and in rare cases surface CD3. However, if more than 75% of leukemic cells express CD5 but retain the other immunophenotypic criteria, the leukemia in this case is called near ETP-ALL [1,3]. CD123 and CD38 may also be expressed on these lymphoblasts more frequently than other T-ALL subtypes [5,7,8].

The immunophenotypic profile of ETP-ALL makes it possible to differentiate this entity from other subtypes of T-ALL (pro-T-ALL, pre-T-ALL, cortical T-ALL and mature T-ALL), which according to the European Group for the Immunological Characterization of Leukaemias. The classification does not express myeloid markers or those of immaturity [9]. It also makes it possible to differentiate it from acute undifferentiated leukemias which by definition lack the T-cell and myeloid markers cCD3 and MPO and do not express B-cell markers such as cCD22, cCD79a, or strong CD19 [1].

Furthermore, T/M-MPAL is characterized phenotypically by the expression of either CD3 and MPO or at least two markers of monocytic differentiation (CD11c, CD14, CD64, lysozyme) [1]. Therefore, to confirm the T lymphoid nature of ETP-ALL lymphoblasts, it is recommended to use cytoplasmic CD3, CD7 and MPO according to the recommendations of “The Italian Association of Pediatric Hematology and Oncology-Berlin Frankfurt Munich” [10]. 

Thus, to confirm CD3 positivity, at least 20% of the blast population must express it with an intensity comparable to the CD3 expressed on residual T lymphocytes using an internal negative control (for example, B lymphocyte) and an internal positive control (residual T lymphocytes) [11]. Surprisingly, rare cases of ETP-ALL with co-expression of B lymphoid markers have been reported in the literature, the clinical significance of which is not well elucidated [12].

In addition, several scoring systems have been proposed for the diagnosis of ETP-ALL. Indeed, in a study carried out by “Tokyo Children’s Cancer Study Group” two scoring systems have been used in order to be able to differentiate ETP-ALL from other subtypes of T-ALL. The first scoring system contains six markers (CD5, CD8, CD13, CD33, CD34, HLA-DR) with 100% specificity and 76.5% sensitivity. The second scoring system contains, in addition to the six markers mentioned above, a more extensive panel by adding CD2, CD3 surface, CD4, CD10, CD56 so that we obtain a combination of 11 markers with a specificity of 100% and a sensitivity of 94.1% (Tables 1-2) [4].

**Table 1:** Scoring system based on the expression of six markers (CD5, CD8, CD13, CD33, CD34, and HLA-DR). ETP-ALL: early T-cell precursor acute lymphoblastic leukemia

Score	-1	+1
CD5	≥5% positive	<75% positive
CD8	≥5% positive	<5% positive
CD13	-	≥25% positive
CD33	-	≥25% positive
CD34	-	≥25% positive
HLA-DR	-	≥25% positive
A score > 4 define ETP-ALL		

**Table 2:** Scoring system based on the expression of 11 markers (CD5, CD8, CD13, CD33, CD34, HLA-DR, CD2, surface CD3, CD4, CD10, and CD56). ETP-ALL: early T-cell precursor acute lymphoblastic leukemia

Score	-2	-1	+1	+2
CD5	≥75%	-	-	<75% positive
CD8	≥5% positive	-	-	<5% positive
CD13	-	-	≥25% positive	≥75% positive
CD33	-	-	≥25% positive	≥75% positive
CD34	-	-	≥25% positive	≥75% positive
HLA-DR	-	-	≥25% positive	≥75% positive
CD2	-	≥75% positive	<20% positive	-
CD3 surface	-	≥75% positive	<20% positive	-
CD4	-	≥75% positive	<20% positive	-
C10	-	≥75% positive	<20% positive	-
CD56	-	-	≥20%	-
A score > 7 define ETP-ALL				

However, this multitude of available scoring systems combined with the technical difficulties of flow cytometry (standardization, etc.) and the variability in the interpretation of the results of this examination make the diagnosis of this entity, solely by flow cytometry, complicated.

On the genetic level, several studies have investigated the genomic landscape of ETP-ALL. They concluded that this entity presents a heterogeneous mutational status without specific genetic abnormality [13]. Molecular and cytogenetic abnormalities observed during typical T-ALL are rarely detectable or even absent in this entity, in particular, NOTCH1, FBXW7 mutations and deletions of CDKN2AB [5,13-15].

On the other hand, a higher frequency of mutations typically associated with AML has been reported, particularly those activating mutations in genes regulating cytokine receptor and RAS signalling (FLT3, JAK1, JAK3, KRAS, NRAS, IGFR1, IL7r, SH2B3, BRAF), those which affect genes coding for transcription factors (ETV6, RUNX1, GATA3, IKZF1, HOXA, EP300), as well as mutations which affect histone-modifying genes (EED, EZH2, SUZ12, DNMT3A). The high frequency of MEF2C and KMT2A rearrangements is also noted [5,13,14,16].

Given all these data, the diagnosis and management of this hemopathy constitutes a challenge for the biologist and clinician. Indeed, several old studies carried out in the pediatric population followed for ALL, whose treatment was based on standard chemotherapy regimens, have shown a poor outcome of patients with more frequent induction failure and relapse/refractory disease of ETP-ALL in comparison with other subtypes of ALL. This can be explained by a very heterogeneous genomic landscape of ETP-ALL [3,13,17,18]. However, treatment regimens using intensive chemotherapy with allogenic hemopoietic stem cell transplantation, and based on MRD monitoring, have been able to improve the prognosis of this hemopathy [13,17].

Recently, with the improved understanding of the pathophysiology and the genomic features of this entity, several novel therapies (targeted therapies), used alone or combined with other therapeutic regimens, are being developed for ETP-ALL. The BCL2 inhibitor (venetoclax), JAK inhibitor (ruxolitinib), FLT3 inhibitors and immunotherapies, including CAR-T, CAR-NK and both monoclonal antibodies and immunoconjugates targeting CD38, CD33 and CD123, are among the therapeutic options available [7,13,18].

## Conclusions

ETP-ALL is a unique and uncommon form of T-ALL. It represents a significant clinical challenge due to its heterogeneous mutational landscape and poor response to conventional therapies. This subtype is defined by a distinct immunophenotypic profile that includes the expression of myeloid and stem cell markers, making it challenging to distinguish from other leukemias. Recent advancements in understanding the genomic features of ETP-ALL have underscored the need for tailored therapeutic approaches.

While traditional chemotherapy regimens have shown limited efficacy, emerging treatments - including targeted therapies and intensive chemotherapy followed by allogeneic hematopoietic stem cell transplantation - offer hope for improved outcomes. Additionally, the development of scoring systems for accurate diagnosis emphasizes the importance of a multidisciplinary approach in managing this condition. Overall, more in-depth studies are necessary to better understand the clinical-biological characteristics of ETP-ALL, namely the genetic particularities. Indeed, with the advent of new generation technologies, other secrets hidden by this entity can be revealed, thus allowing better management of this hemopathy based on personalized care.
